# ZC3H11A loss of function enhances NF-κB signaling through defective IκBα protein expression

**DOI:** 10.3389/fimmu.2022.1002823

**Published:** 2022-11-09

**Authors:** Mahmoud Darweesh, Shady Younis, Zamaneh Hajikhezri, Arwa Ali, Chuan Jin, Tanel Punga, Soham Gupta, Magnus Essand, Leif Andersson, Göran Akusjärvi

**Affiliations:** ^1^ Department of Medical Biochemistry and Microbiology, Uppsala University, Uppsala, Sweden; ^2^ Department of Microbiology and Immunology, Faculty of Pharmacy, Alazhr University, Assiut, Egypt; ^3^ Division of Immunology and Rheumatology, Stanford University, Stanford, CA, United States; ^4^ Department of Immunology, Genetics and Pathology, Uppsala University, Uppsala, Sweden; ^5^ Division of Clinical Microbiology, Department of Laboratory Medicine, Karolinska Institutet, Stockholm, Sweden; ^6^ Department of Animal Breeding and Genetics, Swedish University of Agricultural Sciences, Uppsala, Sweden; ^7^ Department of Veterinary Integrative Biosciences, Texas A&M University, College Station, TX, United States

**Keywords:** ZC3H11A, NF-κB, IκBα, IL6, adenovirus

## Abstract

ZC3H11A is a cellular protein associated with the transcription export (TREX) complex that is induced during heat-shock. Several nuclear-replicating viruses exploit the mRNA export mechanism of ZC3H11A protein for their efficient replication. Here we show that ZC3H11A protein plays a role in regulation of NF-κB signal transduction. Depletion of ZC3H11A resulted in enhanced NF-κB mediated signaling, with upregulation of numerous innate immune related mRNAs, including IL-6 and a large group of interferon-stimulated genes. IL-6 upregulation in the absence of the ZC3H11A protein correlated with an increased NF-κB transcription factor binding to the IL-6 promoter and decreased IL-6 mRNA decay. The enhanced NF-κB signaling pathway in ZC3H11A deficient cells correlated with a defect in IκBα inhibitory mRNA and protein accumulation. Upon ZC3H11A depletion The IκBα mRNA was retained in the cell nucleus resulting in failure to maintain normal levels of the cytoplasmic IκBα mRNA and protein that is essential for its inhibitory feedback loop on NF-κB activity. These findings indicate towards a previously unknown mechanism of ZC3H11A in regulating the NF-κB pathway at the level of IkBα mRNA export.

## Graphical Abstract

ZC3H11A depletion resulted in the retaining of IkBa mRNA in the cell nucleus, failing to maintain normal levels of the cytoplasmic IkBa mRNA and protein that is essential for its inhibitory feedback loop on NF-kB activity.

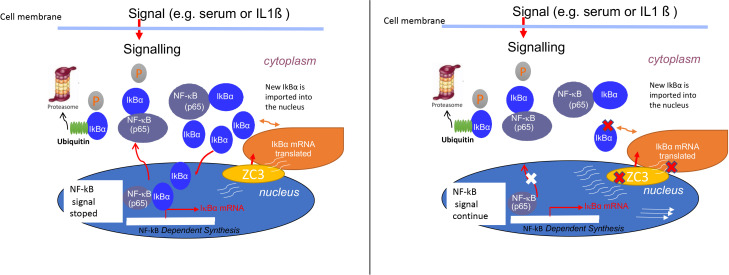

## Introduction

NF-κB is a ubiquitous transcription factor that controls the expression of a large group of genes involved in cell proliferation, inflammation, and survival (1). The NF-κB signaling pathway is subjected to a complex regulation, essential for the fine-tuned physiological response of cells to extracellular and intracellular stimuli (2, 3). Uncontrolled innate immune responses activated through NF-κB signaling may lead to exaggerated activation of NF-κB downstream targets, which may have harmful effects on the host and cause severe inflammatory diseases (4–8).

The NF-κB transcription factor consists of a dimer of proteins that belongs to the NF-κB/Rel family of proteins (p50, p52, p65, c-Rel, and RelB) (9, 10). The p65-p50 heterodimer, which represents the most prevalent form of NF-κB, is expressed in nearly all cell types (9, 11). In unstimulated cells, this complex is present in an inactive cytoplasmic trimeric complex together with the inhibitor of NF-κB (IκB) protein, such as the IκBα protein. Stimulation of cells with TNF-α, IL-1ß, or LPS leads to an activation of a signaling cascade that results in phosphorylation and ubiquitination of IκB, which in turn leads to proteasomal degradation of the IκB protein (12, 13). The released NF-κB transcription factor migrates to the cell nucleus, where it binds to specific NF-κB DNA binding sites and activates transcription of a large number of downstream genes (14, 15).

Transcription of the IκBα protein, which is the best studied member of the IκB family of inhibitory proteins, is in itself under the control of the NF-κB transcription factor, which leads to a negative feedback loop controlling NF-κB activity (16, 17). After synthesis, the IκBα protein is transported to the cell nucleus where it binds to NF-κB, leading to a conformational change that loosens the DNA binding activity of NF-κB. The newly reformed trimeric complex is then exported to the cytoplasm, where it stays inactive until a new round of activation occurs (18). Quantitative models of cell signaling suggest that the time and speed of NF-κB inactivation critically depend on the concentration of the negative feedback loop components (19, 20). It has also been shown that the rate of NF-κB and IκBα synthesis and degradation determine the magnitude and extent of NF-κB signaling (14). These studies have collectively highlighted the importance of IκBα as a major player that controls the NF-κB signaling pathway. The free IκBα protein is highly unstable, with a half-life of less than 10 min whereas IκBα-complexed to NF-κB is highly stable, with an *in vivo* half-life of several hours (21, 22).

The human genome codes for at least 57 CCCH-type of zinc finger (ZnF) proteins (23, 24). The majority of these are involved in the regulation of RNA metabolism (e.g., RNA splicing, polyadenylation, mRNA export, and translation). Interestingly several CCCH-type ZnF proteins have also been shown to be involved in inflammation, immune homeostasis, and immune cell maturation (25–27). Well studied examples are TTP (also known as ZFP36) (28), Roquin 1 (29) (also known as RC3H1), and ZC3H12A (also known as MCPIP1) (30, 31). These ZnF proteins appear to destabilize several cytokine mRNAs involved in inflammation, innate and adaptive immune responses, and immune system homeostasis maintenance (32–34).

One of the enigmatic CCCH-type ZnF proteins is ZC3H11A. Previous proteomic studies have shown that ZC3H11A is a component of the Transcription-Export (TREX) complex, a multiprotein complex that serves a key function in nuclear mRNA export (35, 36). The ZC3H11A protein is dispensable for HeLa cell growth under normal conditions. However, CRISPR/Cas9 ZC3H11A HeLa knock-out (ZC3-KO) cells show a retarded growth after heat-shock, suggesting that ZC3H11A is a stress-induced protein (37). Interestingly, we have shown that several nuclear replicating human viruses (HAdV-5, HIV-1 HSV-1, and influenza virus) were impeded in their growth in ZC3-KO cells (37). Mechanistically, the absence of the ZC3H11A protein caused a nuclear retention of some of the HAdV-5 mRNAs suggesting a role for ZC3H11A in nuclear export of specific mRNAs.

Here we have expanded our previous work by examining the effect of ZC3H11A on the expression of immune response genes. In contrast to the requirement of ZC3H11A for efficient viral late protein expression, the loss of ZC3H11A protein expression stimulated IL-6 protein expression, and enhanced accumulation of a large group of immune response mRNAs. Our data show that the enhanced accumulation of IL-6 was due to an upregulation of the NF-κB signaling pathway in ZC3-KO cells. Mechanistically, the enhancement of the NF-κB signaling pathway correlated with a defect in IκBα inhibitory mRNA and protein accumulation. Our results suggest that ZC3-KO cells fail to produce normal levels of the cytoplasmic IκBα mRNA, thereby failing to produce sufficient amounts of the IκBα protein to maintain the inhibitory feedback loop on NF-κB signaling (**Graphical abstract**).

## Materials and methods

### Cell culture and plasmid construction

Human cervical carcinoma HeLa cells [American Type Culture Collection (ATCC) CCL-2] were maintained in Dulbecco’s modified Eagle medium (DMEM) with 2 mM L-glutamine, 1 mM sodium pyruvate, and 4.5 g/L glucose (ATCC 30-2001), supplemented with 10% heat-inactivated fetal bovine serum (FBS) and penicillin (0.2 U/mL)/streptomycin (0.2 μg/mL) (Gibco) at 37°C in a 5% CO2 humidified atmosphere. The HeLa ZC3-KO cell line has previously been described (37). The HeLa control cells are the same stock as previously used to construct the HeLa ZC3-KO cells (37). The NF-κB pathway was stimulated in cells by treatment with recombinant human IL-1ß (Gibco) at a final concentration of 10 pg/mL. The pGFP-IL-6 3’ UTR plasmid was constructed by cloning a 429 base-pair fragment, starting immediately after the IL-6 stop codon (ENSE00001552940; 22,731,573 – 22,732,002), into a pEGFP-C2 vector using XhoI and XbaI restriction enzyme cleavage sites.

### Actinomycin D chase assay

HeLa or ZC3-KO cells were stimulated with 10 pg/mL IL-1ß for 30 min followed by a treatment with 10 µg/mL actinomycin D (Wako) for 2 h. Samples were collected at 0, 30 min, and 2 h post-actinomycin D addition. Total RNA was isolated from the collected cells using the TRI reagent (Sigma). IL-6 mRNA expression was analyzed using northern blot and RT-qPCR.

### RNA-sequencing

Triplicate samples of HeLa cells subjected to ZC3 siRNA or scrambled siRNA knockdown were subjected to RNA-Seq analysis. In brief, 48 h post knockdown, around 4 million cells were washed in PBS, and total RNA extracted using the RNeasy Mini Kit. The RNA quality and integrity were verified with an RNA ScreenTape assay (TapeStation; Agilent Technologies). Strand-specific mRNA-seq libraries were generated using a SENSE RNA-Seq Library Prep Kit (Lexogen), following the manufacturer’s instructions. For each sample, 2 μg of total RNA was enriched for mRNA by polyA-selection using oligo(dT) beads, and the RNA-seq libraries amplified by 12 PCR cycles. The libraries were sequenced as 125-bp paired-end reads using Illumina Nova-seq. The RNA-seq reads have been submitted to the sequence read archive (http://www.ncbi.nlm.nih.gov/sra) with the accession numbers (to be added once the manuscript has been accepted).

### Bioinformatic analyses

Sequence reads were mapped to the reference human genome (hg38) using STAR 2.5.1b with default parameters (38). HTSeq-0.6.1 (Python Package) (39) was used to generate read counts and edgeR (Bioconductor package) (40) was used to identify differentially expressed genes using gene models and annotation GTF file for hg38 downloaded from UCSC (www.genome.ucsc.edu). The abundance of gene expression was calculated as count-per-million (CPM) reads. Genes with less than three CPM in at least three samples were filtered out. The filtered libraries were normalized using the trimmed mean of M-values (TMM) normalization method (41) *P*-values were corrected for multiple testing using the False Discovery Rate (FDR) approach. For gene ontology analysis, the differently expressed genes were analyzed using the Clusterprofiler R package (42). All expressed genes were used as background, and the Biological Process pathway tables were used to identify enriched GO terms. The gene set enrichment analysis (GSEA) was performed using the fgsea R package (43). The genes were ranked based on the fold-change (siZC3/scrambled) and the datasets were downloaded from the GSEA website (https://www.gsea-msigdb.org/gsea/downloads.jsp). The RNA-seq reads have been submitted to the short reads archive (http://www.ncbi.nlm.nih.gov/sra) with the accession numbers (to be added once the manuscript has been accepted).

### siRNA knockdown

Transfection of siRNA was done using the JetPrime transfection reagent (Polyplus) according to the manufacturers protocol. The sequence of the siRNAs used in this study are listed in **Table S1**.

### Immunoblot analysis

Total protein lysates were prepared using RIPA lysis buffer (150 mM NaCl, 50 mM HEPES [pH 7.4], 0.5% sodium deoxycholate, and 0.1% SDS) containing protease inhibitors (Complete Ultra Tablets, Roche). Equal amounts of total lysates were separated on a gradient SDS-PAGE (AnyKd, Bio-Rad) and transferred to a PVDF membrane (Millipore). Intercept^®^ blocking buffer (LI-COR) was used to block the membrane before the indicated primary and secondary antibodies were used for immunodetection. The following primary antibodies were used: phospho-IκBα (Ser32), total IκBα, GFP, actin, Lamin B1, HAdV capsid, tubulin (see **Table S2** for details). Following an overnight incubation, the membrane was washed with 1x Tris-buffered saline with 0.1% Tween-20 (TBST) and incubated with the IRDye^®^ secondary antibodies (LI-COR). Membranes were visualized using the Odyssey CLx imaging system (LI-COR).

### RNA-FISH

To detect the IκBα mRNA we used a 70-nucleotide long HPLC-purified oligonucleotide probe labeled at the 5’ end with Alexa 488 (**Table S1**). The probe was complementary to the coding sequence of the IκBα mRNA (Genebank Accession: AK097828.1). Cells were grown on glass slides overnight. The next day cells were fixed with 4% paraformaldehyde for 10 min, washed three times with PBS, permeabilized with 0.5% Triton X-100 for 10 min at room temperature, and rinsed once with PBS. The hybridization buffer [0.4 μM oligonucleotide probe, 0.5 μg of transfer RNA (Ambion), 1% BSA, 10% (vol/vol) dextran sulfate, 50% deionized formamide, and 2x sodium saline citrate (SSC)] was added to the cells and incubated overnight at 37°C in a chamber containing 50% deionized formamide and 2 x SSC as a humidifier solution. The following day cells were washed twice with 2 x SSC, once with 0.5 x SSC, and once with PBS before visualized in a Nikon i90 Upright microscope.

### Electrophoretic mobility shift assay (EMSA)

EMSA were performed according to the previously published protocol (44). A HeLa cell nuclear extract was prepared using the NucBuster™ Protein Extraction Kit (Novagen). About 10^6^ cells per sample were collected and resuspended in 75 µl NucBuster Reagent 1 buffer to lyse the cytoplasmic membrane. The nuclear pellet was resuspended in 75 µl NucBuster Extraction Reagent 2, containing 1 μl 100x Protease Inhibitor Cocktail, and 1µl of 100 mM DTT. After centrifugation the supernatant was collected and labeled as nuclear extract and stored at -80°C. The oligonucleotide sequences containing the transcription factor binding sites assayed in this study are listed in **Table S1**. The double-stranded oligonucleotides were 5’ end labelled using γ- (32)P-ATP and purified as previously described (45). The nuclear extract (4 µl) was incubated with the (32) P-labelled probe (3 µl, 0.05 pmol) in 5 µl of 4x binding buffer, 1 µl Poly(dI-dC), and 7 µl water for 30 min. As a cold competitor 1 µl containing a 10-fold excess of the unlabeled double-stranded oligonucleotide was added to the binding buffer. All samples were loaded and resolved on a 5% native polyacrylamide gel. The result was visualized by exposure of membranes to a PhosphorImager screen (Fuji) followed by signal analysis using the Image One software (BioRad).

### Reporter assays

Hela cells were seeded in 6 well plate at 500 000 cells/well. The next day cells were transfected with a ZC3H11A siRNA or a scrambled control siRNA. 24 h post siRNA transfection cells were transfected with the pSIRV-NF-kB-eGFP reporter plasmid [contains a minimal NF-kB responsive promoter (Addgene 118093)] or the PeGFPc1 plasmid expressing eGFP under the control of the CMV promoter. Cells were collected 48 h post transfection and GFP expression quantitated by Western blot analysis.

### Monocytes and monocyte derived dendritic cells

Peripheral blood mononuclear cells (PBMCs) were isolated by Ficoll-Paque (GE Healthcare Life Science) from fresh buffy coats of healthy anonymized donors, collected at the Blood Center at Uppsala University Hospital. PBMCs were cultured in RPMI-1640 supplemented with 10% FBS, 1 mM sodium pyruvate, and 1% Penicillin/Streptomycin. CD14+ cells in the PBMC pool were isolated by using specific beads (Miltenyi Biotec) and transduced with lentivirus pBMN(GFP-shRNA) (46). The shRNA was designed to knockdown the human *ZC3H11A* gene and synthesized by IDT (Integrated DNA Technologies). The sequence of the shRNA used is shown in **Table S1**. The shRNA was subcloned into pBMN lenti-plasmid and lentivirus produced as previously described (47) The transduced monocytes were differentiated over 5 days into immature DCs (imDCs) in the presence of 50 ng/mL GM-CSF (Gentaur) and 25 ng/mL IL-4 (Gentaur). Fresh medium with cytokines was replaced every 2 day. Phenotypic analysis of human DCs with their surface marker expression was performed using flow cytometry BD Canto II (BD Biosciences). The markers examined were: CD14, CD11c, CD80 and CD86. Data analysis was performed using Flowjo (BD Biosciences). ImDCs were stimulated with IL-1β (Nordic BioSite AB, 10 ng/mL) and TNF-α (Nordic Biosite AB, 10 ng/mL) and IL-6 production was measured with ELISA (Mabtech) from the culture supernatant.

### RNA extraction and RT-qPCR

Total cytoplasmic RNA was extracted by lysing cells in IsoB-NP-40 buffer [10 mM Tris–HCl (pH 7.9), 150 mM NaCl, 1.5 mM MgCl_2_ and 1% Nonidet P-40], removal of the nuclear pellet, followed by two rounds of phenol-chloroform–isoamyl alcohol extraction and one extraction with chloroform–isoamyl alcohol, as described previously (48). Total RNA was extracted using the TRI reagent (Sigma) according to the manufacturer’s instructions. The cDNA synthesis was performed using the SuperScript III Reverse Transcriptase kit according to the manufacturer’s instructions (ThermoFisher). Quantitative RT-PCR was performed on an ABI Fast Real-Time PCR system (Applied Biosystems) using HOT FIREPol^®^ EvaGreen^®^ qPCR Mix Plus kit from Solis Biodyne under the following cycling conditions: 94°C, 5 min, once; 94°C, 15 s, 60°C, 20 s, 72°C, 20 s, 40 cycles. Forward and reverse primers were mixed with the HOT FIREPol^®^ EvaGreen^®^ qPCR Mix in a 20 µl total reaction volume. The quality of PCR products was examined by electrophoresis on a 1% agarose gel. The primers used in the PCR reactions are listed in **Table S1**.

### Northern blot

RNA was loaded onto a denaturing 1% agarose gel and blotted to a Hybond NX membrane (Amersham Biosciences), cross-linked at 80°C for 1 h, and hybridized as previously described in detail (49). Hybridization probes were generated by 5′-end labeling with γ- (32) P-ATP DNA oligonucleotides complementary to the 5’ end of either the coding sequence of the IL-6 mRNA, or the coding sequence of IκBα. Signals were detected by exposure of membranes to a PhosphorImager screen (Fuji) followed by signal analysis using the Image One software (BioRad). The sequence of the oligonucleotides used for hybridization are listed in **Table S1**.

## Results

### ZC3H11A is a regulator of innate immune signaling

**Figure 1:**
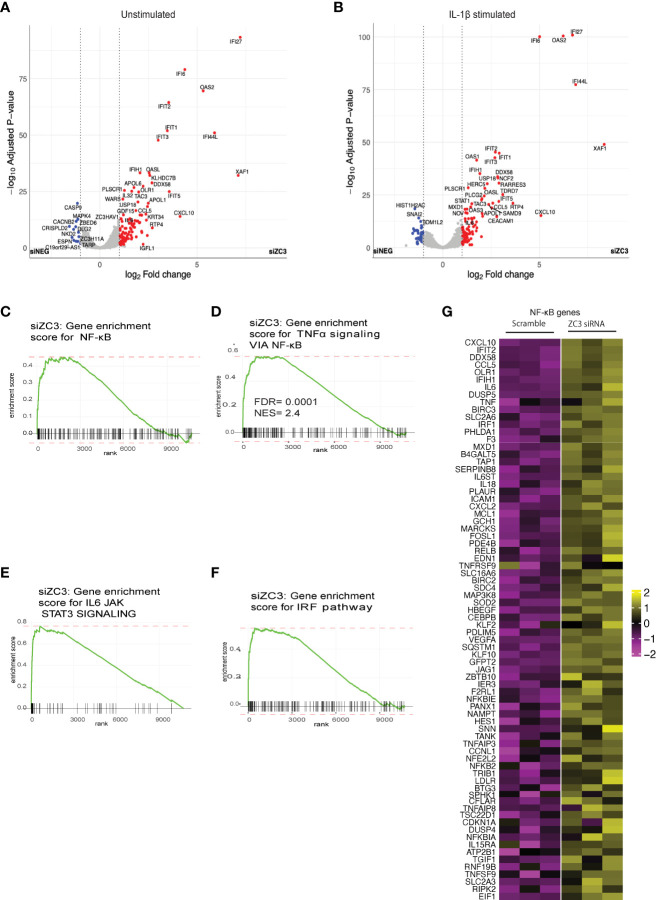
ZC3H11A is a regulator of innate immune signaling. Volcano plot analysis of the cellular mRNA expression profile in unstimulated **(A)** and IL-1b stimulated **(B)** HeLa cells transfected with a ZC3H11A (siZC3) or a scrambled negative control (siNEG) siRNA. Data points represent the log2 fold-changes of the triplicate experiments (n = 3 per group). Blue, down-regulated (Padj<0.05); grey, non-regulated; red, upregulated (Padj<0.05) mRNAs. **(C-F)** Gene set enrichment analysis of ranked DE genes in ZC3-KD cells using hallmark gene sets. **(G)** Heatmap of the genes contributing to TNF-a signalling via NF-kB pathway.

Our previous studies have shown that ZC3H11A is a stress-induced protein that is involved in nuclear export of mRNA. Thus ZC3H11A can play a key role in translational control of gene expression and in turn cellular signaling pathways. In order to determine the cellular signaling pathways that is regulated by ZC3H11A, we used RNA-seq to analyze the cellular mRNA expression profile in HeLa cells where ZC3H11A levels had been knocked down by using siRNA (**Figure S1A**). The abundance of gene expression was calculated as count-per-million (CPM) reads. In ZC3H11A siRNA knockdown (ZC3-KD) cells compared to the siRNA scrambled control, we detected more than 900 differentially expressed genes (adjusted P-value <0.05), of which more than 28 exhibited a ≥2-fold change and majority showing an upregulation antiviral immune response pathways (**Figures 1A**). In order to determine how the immune response pathways are regulated by ZC3 upon immunological stress, we treated the ZC3-KD and siRNA control HeLa cells with IL-1ß and performed RNA-seq showing a similar trend (**Figure 1B**). The gene set enrichment analysis (GSEA) of ranked DE genes in ZC3-KD cells using the hallmark gene sets revealed a positive enrichment of genes involved in the IL6-JAK-STAT3 signaling and TNF-α and NF-κB pathways (**Figures 1C-F** and **Figure S1B**). Furthermore, the GSEA using the sets of transcription factor targets revealed a significant enrichment of genes having at least one occurrence of the motif “GGGAMTTYCC” matching the NF-κB transcription factor binding site (**Figure 1C**) or the motif “BNCRSTTTCANTTYY” matching the IRF1 transcription factor binding site (**Figure 1F**), located in the regions spanning 4k base pairs centered on their transcription start sites [-2k bp, +2k bp]. The heatmap (**Figure 1G**) present the expression of the subset of genes that contributed the most to the enrichment signals in TNF-α signaling *via* the NF-κB pathway.

While we did not observe an increased mRNA expression of ZC3H11A in IL-1ß treated HeLa cells (**Figure S1C**), TNF-α stimulation resulted in a significant increase in the level of ZC3H11A mRNA (**Figure S1D**) suggesting towards a NF-κB dependent ZC3H11A gene expression.

### ZC3H11A negatively regulates NF-κB signaling

**Figure 2:**
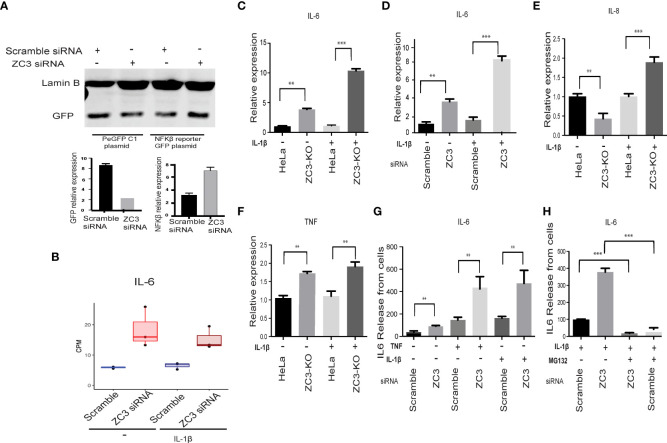
ZC3H11A negatively regulates NF-κB signaling. A GFP reporter contains a minimal NF-kB responsive promoter transfected to HeLa treated with scramble or ZC3 siRN. The expression of GFP from the GFP control plasmid and the GFP- NF-kB responsive promoter plasmid was visualized by western blot **(A)**. Expression of IL-6 mRNA in RNA seq normalized to GAPDH **(B)**. ZC3-KO or HeLa wild type cells transiently transfected with siRNAs were treated with IL-1ß (10 pg/ mL) for 1h. Total cytoplasmic RNA was isolated for RT-qPCR analysis of relative expression of IL-6 in treated or untreated ZC3-KO and HeLa wild type cells **(C)** or ZC3-KD and scrambled siRNA transfected cells **(D)**. Relative expression of IL-8 **(E)** and TNF were also quantitated in the ZC3-KO cells **(F)**. The release of IL-6 into the supernatant of HeLa cells transfected with a ZC3H11A or a scrambled siRNA was measured by an ELISA assay after 1 h of IL-1ß (10 pg/ mL or TNF (10 ng/ mL) treatment. **(G)**. Pretreatment of cells with MG132 (5 µM) for 3 hrs before IL-1ß stimulation blocked NF-κB signaling and IL6 release **(H)**. Data representative of three individual experiments with n = 3 technical replicates per treatment. **p < 0.01, ***p < 0.001, as determined by ordinary one-way Anova. All error bars represent ± SDs.

Earlier studies on another CCCH domain containing Zinc finger, ZC3H12A have shown its inhibitory effect on activation of NFκB (50). Since in our transcriptomics data we have observed an enrichment of genes associated with NF-κB pathways upon knockdown of ZC3H11A, we asked whether ZC3H11A has any regulatory effect on transcriptional activation of NF-κB. To this end we made use of an eGFP reporter plasmid driven by a minimal NF-κB responsive promoter element (51). As a control we used an eGFP reporter under the transcriptional control of the CMV promoter. Cells were transfected with a ZC3-siRNA or scramble control siRNA. 24 h post siRNA transfection cells were transfected with the eGFP reporter plasmids. Following a further 48 h incubation the GFP signals were quantified using Western Blot and densitometry. The GFP signal in ZC3H11A knockdown cells was almost double that of cells treated with the control scramble siRNA (**Figure 2A**). While the GFP signal from the plasmid expressing GFP from the CMV promoter was unaffected by ZC3H11A knockdown. This result indicates that the ZC3H11A protein suppresses the activity of the NF-κB transcription factor.

IL-6 mRNA expression is known to be under the transcriptional control of NF-κB (52). In our RNAseq data we noted an increase in IL-6 mRNA expression in ZC3-KD cells (**Figure 2B**). To test the hypothesis that the NF-κB pathway is overactive in cells lacking ZC3H11A we analyzed NF-κB downstream genes IL-6, IL-8, and TNF-α mRNA expression in untreated and IL-1ß stimulated HeLa wild-type and ZC3-KO HeLa cells (37) by RT-qPCR. As shown in **Figure 2C**, IL-6 mRNA expression was almost 4-fold higher in ZC3-KO cells compared to wild-type cells, which was further amplified to nearly 10-fold when the NF-κB pathway was stimulated with IL-1ß. Similar IL-6 upregulation was also observed in ZC3-KD cells (**Figure 2D**). Alike to IL-6, TNF-α mRNA expression was enhanced in ZC3-KO cells (**Figure 2F**), although not to the same extent as IL-6. However, In contrast to IL-6, expression IL-8 mRNA expression was markedly reduced in unstimulated ZC3-KO cells (**Figure 2E**) that significantly increased upon IL-1ß stimulation (**Figure 2E**), although not to the same extent as IL-6 (**Figures 2C, D**).

We also quantitated IL-6 protein expression in the supernatant of IL-1ß and TNF-α stimulated or unstimulated ZC3H11A-siRNA transfected cells using ELISA. There was an increased IL-6 release by the ZC3-KD cells that was further enhanced upon stimulation of the NF-κB pathway with IL-1ß or TNF-α (**Figure 2G**). NF-κB activation is dependent on the K48-linked ubiquitination and proteosomal degradation of its inhibitory IκBα protein (53). When the IL-1ß stimulated cells were treated with proteosomal inhibitor MG132, IL-6 release was significantly diminished in ZC3-KD cells (**Figure 2H**) further concluding that the enhanced IL-6 production in ZC3-KD cells is due to NF-κB transcriptional activity.

### Downregulation of ZC3H11A induces human monocyte maturation into dendritic cells and IL-6 expression in the immature dendritic cells

**Figure 3:**
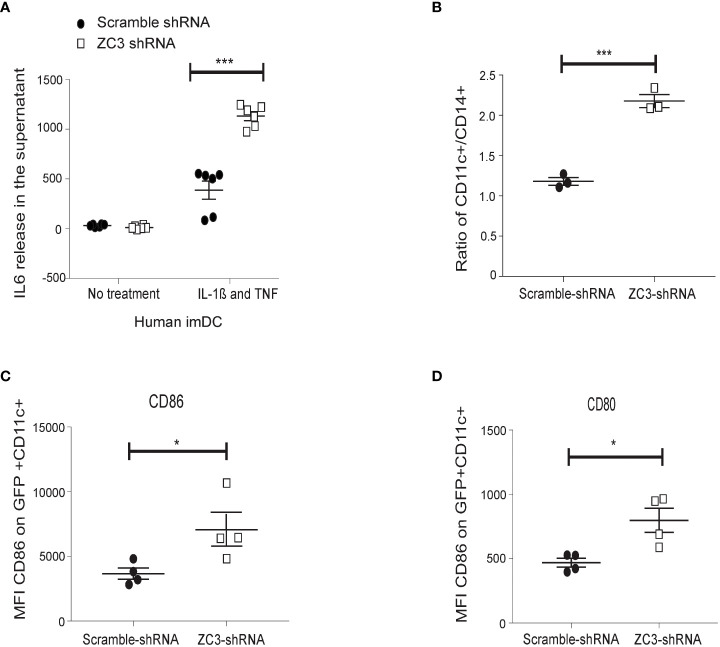
ZC3H11A depletion induces IL-6 expression in primary human dendritic cells, and enhances monocytes maturation into dendritic cells. **(A)** CD14+ monocytes were isolated by positive selection from PMBCs. Cells were transduced with a lentivirus expressing an shRNA-targeting ZC3H11A, or a lentivirus expressing a nonspecific scrambled shRNA. IL-6 release was measured by ELISA from cells treated or not treated with an IL-1ß and TNF cocktail. The enhancement of dendritic maturation marker expression in the lentivirus-infected cells were followed by flow cytometry; CD11c **(B)**, CD86 **(C)**, and CD80 **(D)** marker expression. The data are presented as the mean ± SEM values. *P < 0.05; ***P < 0.001.

We used a lentivirus expressing a ZC3H11A short-hairpin RNA (shRNA) to knockdown ZC3H11A expression in primary human monocytes to validate our findings from the HeLa cell model system. As shown in (**Figure 3A**), ZC3-KD resulted in a significantly higher release of IL-6 in the supernatant after stimulation with a cocktail of IL-1ß and TNF-α compared to the lentivirus expressing a scrambled shRNA. We further noted that the ratio of CD11c+ and CD14+ cells increased in ZC3-KD cells compared to the scrambled shRNA treatment (**Figure 3B**). Since CD11c+ is a marker for immature dendritic cells (imDCs), this result suggests that ZC3-KD promoted monocyte (CD14+) differentiation towards imDCs. We also noted that the co-stimulatory molecules, CD80 and CD86, which are NF-κB target genes, were significantly upregulated in ZC3-KD cells (**Figures 3C, D**). These results collectively strengthen the conclusion that the loss of the ZC3H11A protein results in an upregulation of NF-κB signaling.

### Mechanism for IL-6 mRNA overexpression in ZC3-KO cells

**Figure 4:**
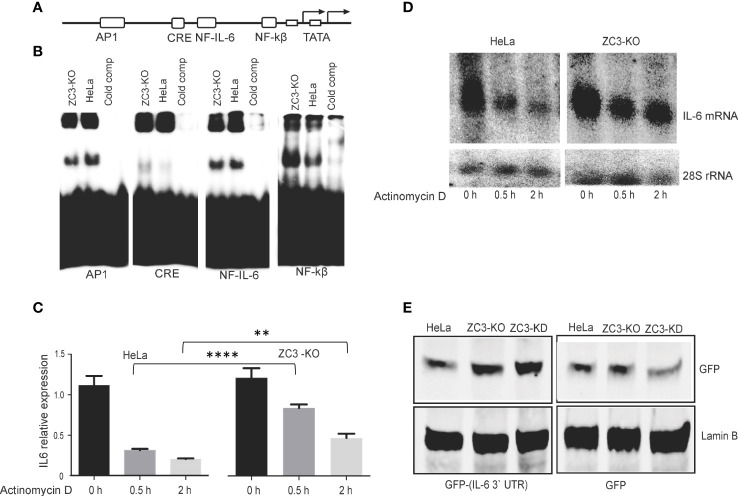
Mechanism for IL-6 mRNA overexpression in ZC3-KO cells. Schematic drawing of the transcription factor binding sites in the IL-6 promoter **(A)**. Nuclear extracts were prepared from HeLa cells or ZC3-KO cells treated with IL-1ß (10 pg/ mL) for 4 hrs. Extracts were incubated with 32P-labeled double-stranded oligonucleotides corresponding to the transcription factor binding sites depicted in panel A (**Table S1**). Complexes were resolved on a neutral agarose gel, dried and visualized on a Fuji PhosphorImager. In lanes labeled “cold comp” the ZC3-KO extract was incubated with a 10-fold excess of the corresponding unlabeled oligonucleotide **(B)**. HeLa cells or ZC3-KO cells stimulated with IL-1ß for 30 min were treated with actinomycin D and samples collected at 0 h, 0.5 h, and 2 h after actinomycin D addition. Total cytoplasmic RNA was isolated and the effect of actinomycin D on IL-6 mRNA stability were quantitated by RT-qPCR, the data are presented as the mean ± SEM values. **P < 0.01; ****p < 0.0001; in unpaired t test **(C)** and visualized by northern blot. 28S rRNA accumulation served as loading control in the northern blot assay **(D)**. A GFP reporter containing the IL-6 3’ UTR was constructed and transfected to HeLa wild type, HeLa-KO or HeLa-KD cells. The expression of GFP from the GFP control plasmid and the GFP-IL-6 3’ UTR plasmid was visualized by western blot **(E)**.

Next, we tested whether the increase in IL-6 mRNA expression in ZC3-KO cells (**Figure 2**) correlated with an increase in transcription factor binding to the IL-6 promoter. For this experiment, nuclear extracts prepared from IL-1ß stimulated HeLa wild-type or ZC3-KO cells were incubated with four different 32P-labeled double-stranded DNA probes, corresponding to the known transcription factor binding sites in the IL-6 promoter (**Figure 4A**), in an electrophoretic mobility shift assay (EMSA). The protein binding activity to the NF-κB and cAMP-response element (CRE)-binding sites were significantly increased in ZC3-KO cells (**Figure 4B**). In contrast, binding to the NF-IL-6 (also known as C/EBP) was unaffected with slightly lower binding of the AP1 binding sites. Based on these experiments we conclude that the lack of ZC3H11A protein expression enhances NF-κB protein binding to its binding site in the IL-6 promoter. This result is compatible with the observations that the increased IL-6 mRNA accumulation in ZC3-KO cells (**Figures 2C, D**), at least in part, may be due to a rise in IL-6 transcription.

A previous study showed that ZC3H11A binds to the SOX resistance element (SRE) present in the 3’ untranslated region (UTR) of the IL-6 mRNA (54). This RNA element binds many cellular proteins, of which a subset protects the IL-6 mRNA from degradation. This observation made it interesting to determine whether ZC3H11A might play a role in IL-6 mRNA stability. Since most RNA-binding proteins that bind to the 3’ UTR control mRNA turnover, we tested the stability of the IL-6 mRNA in ZC3-KO cells. For this experiment HeLa wild-type and ZC3-KO cells were treated with actinomycin D, to block new mRNA synthesis. The stability of the IL-6 mRNA was monitored by relative mRNA expression measured by RT-qPCR (**Figure 4C**) and Northern blot analysis using a probe specific for the IL-6 coding sequence (**Figure 4D**), showing the IL-6 mRNA half-life was substantially increased in ZC3-KO cells compared to wild-type HeLa cells. To further show that the destabilizing effect of ZC3H11A on the IL-6 mRNA was *via* the 3’ UTR we reconstructed a GFP reporter plasmid containing the IL-6 3’ UTR. Transfection of a GFP plasmid, either with or without the IL-6 3’ UTR, into wild-type HeLa, HeLa-KO or HeLa-KD cells demonstrated that in ZC3H11A deficient cells GFP protein expression increased from the reporter with the IL-6 3’ UTR (**Figure 4E**). This result is in line with the conclusion that the destabilizing effect of ZC3H11A on the IL-6 mRNA is mediated through the IL-6 3’ UTR.

Collectively, these experiments suggest that the enhancement of IL-6 mRNA accumulation in cells lacking ZC3H11A results from an increase in IL-6 promoter activity and IL-6 mRNA stability.

### ZC3H11A is required for efficient IκBα mRNA biogenesis

**Figure 5:**
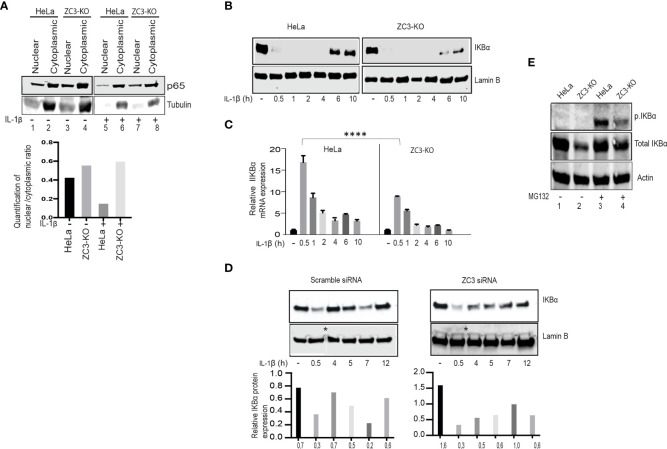
ZC3H11A depletion results in defects in IκBα biogenesis. Western blot analysis of the distribution of the p65 protein in the nuclear and cytoplasmic fractions of HeLa or ZC3-KO cells before and after IL-1ß stimulation **(A)**. Western blot analysis showing the dynamics of total IKBa accumulation after IL-1ß stimulation of HeLa or ZC3-KO cells **(B)**. RT-qPCR experiment showing the relative expression of the IKBa mRNA under the same experimental conditions as in panel C the data are presented as the mean ± SEM values. , ****p < 0.0001; in unpaired t test **(C)**. Western blot analysis showing the dynamics of total IκBα accumulation after IL-1β stimulation in HeLa cells transfected with a scramble control siRNA or a ZC3H11A specific siRNA * represents cropped site and that image came from two different blots are pasted together **(D)**. HeLa or ZC3-KO cells, either treated or not treated with MG132 (5µM, 3 hrs), were probed in a western blot with antibodies detecting phosphorylated or total IκBα **(E)**.

Activation of the NF-κB transcription factor results in a nuclear translocation of the p65/p50 dimer (1). To study NF-κB localization dynamics, we prepared nuclear and cytoplasmic extracts from IL-1ß stimulated HeLa wild-type and ZC3-KO cells. We used western blot analysis to examine the steady-state ratio of the p65 subunit in the two compartments. Interestingly, a larger fraction of the p65 subunit was consistently found in the nuclear fraction of untreated and especially IL-1ß treated ZC3-KO cells compared to HeLa wild-type cells (**Figure 5A**). These results agree with the observation that NF-κB signaling is enhanced in ZC3-KO cells.

In order to monitor the kinetics of NF-κB accumulation, we stimulated HeLa wild-type or ZC3-KO cells with IL-1ß and followed IκBα protein accumulation at different time points post-stimulation (**Figure 5B**). As expected, the IκBα protein expression ceased rapidly after IL-1ß stimulation and was subsequently recovered in HeLa wild-type cells (6 and 10 hrs post-stimulation). The IκBα protein expression was also rapidly lost in ZC3-KO cells. However, it was only partly recovered in the ZC3-KO cells (**Figure 5B**). RT-qPCR quantification of the amount of the IκBα mRNA in the cytoplasm of HeLa wild-type and ZC3-KO cells in the same samples demonstrated a significant reduction in IκBα mRNA expression in ZC3-KO compared to HeLa wild-type cells (**Figure 5C**). Transfection of a ZC3H11A siRNA to HeLa wild type cells mimicked the rapid decline and slow, partial recovery of the IκBα protein expression in ZC3-KD cells (**Figure 5D**).

The upregulation of the NF-κB pathway in ZC3-KO cells could result from a reduction in IκBα protein accumulation because of an increase in IκB kinase activity that is tightly regulated by phosphorylation and ubiquitination (55). To test this possibility, we blocked the proteasomal degradation of IκBα with MG132 in HeLa wild-type and ZC3-KO cells. As shown in (**Figure 5E**), at steady state the level of IκBα was reduced in ZC3-KO cells compared to wild-type cells (lanes 1 and 2). However, after proteasome inhibition, the total IκBα protein level was almost identical in HeLa wild-type and ZC3-KO cells (lanes 3 and 4). The observed increase in IκBα protein levels in ZC3-KO cells after MG132 treatment could indicate towards enhanced ubiquitination of IκBα that could be a reason for increased NF-κB activity. On the other hand MG132 treated ZC3-KO cells showed an observable decrease in the level of the phosphorylated IκBα protein compared to HeLa wild-type cells (lanes 3 and 4), suggesting towards ZC3 regulated feedback mechanism of IκBα activity.

### ZC3H11A is required for efficient nuclear export of the IκBα mRNA

**Figure 6:**
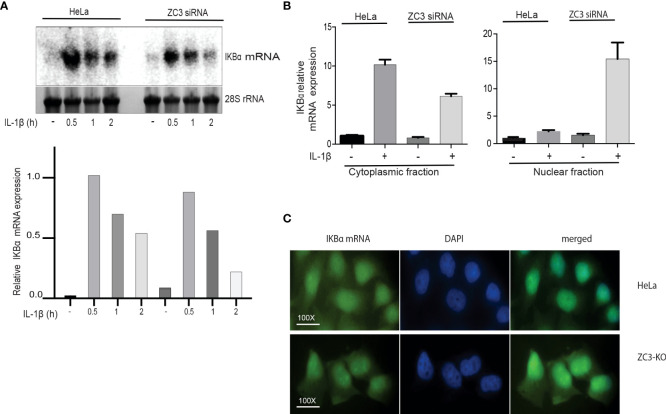
Lack of ZC3H11A expression results in an increased nuclear retention of the IkBa mRNA. Northern blot analysis of IkBa mRNA accumulation in total cytoplasmic RNA isolated from HeLa cells treated with ZC3 siRNA or scramble siRNA cells then treated with IL-1b for three time points. The 28S rRNA served as a loading control **(A)**. RNA from HeLa cells treated with ZC3 siRNA or scramble siRNA treated or untreated with IL-1b were fractionated into nuclear and cytoplasmic fractions. The relative expression of IkBa mRNA was quantitated by RT-qPCR **(B)**. Fluorescent in situ Hybridization (FISH) for Hela cells or ZC3-KO cells using an Alex 488-labeled oligonucleotide probe complementary to the coding sequence of IkBa mRNA **(C)**.

Since we previously showed that ZC3H11A is important for the nuclear export of the adenovirus fiber mRNA we decided to test the effect of ZC3H11A on the nuclear export of the IκBα mRNA. To test this, the level of cytoplasmic IκBα mRNA before and after IL-1ß stimulation was determined by Northern blot analysis in scrambled siRNA and ZC3H11A siRNA transfected HeLa cells and quantitated by densiometry (**Figure 6A**). Similar to the ZC3-KO cells we observed a reduction of the IκBα mRNA in ZC3-KD cells compared to the scramble siRNA-treated cells (**Figure 6A**). To extend the data further, we repeated the siRNA transfection experiment and used RT-qPCR to detect the IκBα mRNA in the nuclear and cytoplasmic compartments of transfected cells. As shown in **Figure 6B**, ZC3-KD resulted in a significant increase in nuclear retention of the IκBα mRNA in IL-1ß treated cells compared to the control cells. At the same time, we observed an almost two-fold drop in IκBα mRNA accumulation in the cytoplasmic fraction (**Figure 6B**). To further confirm this result, we used RNA FISH to determine the localization of IκBα mRNA in ZC3-KO and HeLa wild type cells. The IκBα mRNA showed a higher signal in the nucleus of ZC3-KO cells compared to the wild-type HeLa cells (**Figure 6C**).

Taken together, our results are compatible with a model suggesting that ZC3H11A is important for IκB mRNA transcription in the nucleus and its subsequent export to the cytoplasm.

### Requirement of NF-κB signaling during an adenovirus infection

**Figure 7:**
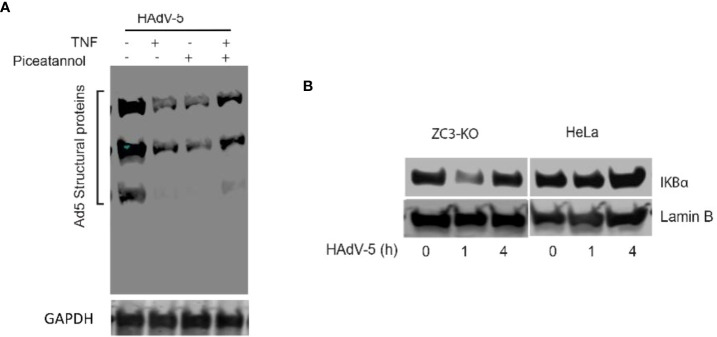
Western blot analysis of adenovirus structural protein expression in Hela cells infected with HAdV-5 and treated with TNF-a and/or Piceatannol **(A)**. Western blot analysis showing the dynamics of IkBa protein expression during the early phase of an HAdV-5 infection (0, 1 or 4 hrs postinfection) in HeLa and ZC3-KO cells **(B)**.

We have earlier shown that Adenovirus grows poorly in ZC3-KO cells (37). In the present study have observed an enhanced NF-κB signaling in both ZC3-KO and ZC3-KD cells. Thus it was tempting for us to speculate that this increase in NF-κB signaling has an inhibitory effect on adenovirus growth. In order to determine this, we pretreated HeLa cells with TNF-α to activate NF-κB signaling, followed by an infection with HAdV-5. As shown in (**Figure 7A**), TNF-α treatment resulted in a drastic inhibition of HAdV-5 late protein expression. A similar inhibition HAdV-5 late protein expression was also observed with pretreatment of the infected cells with an inhibitor of TNF-α mediated NF-κB activation, piceatannol (56), while pretreatment with both TNF-α and piceatannol resulted in a partial recovery of virus replication. This result is compatible with the hypothesis that NF-κB is required for the initial phase of an adenovirus infection and needs to be shut-down at later stages of infection (see Discussion). Further, we observed that IκBα protein levels dropped transiently in ZC3-KO cells at 1 h post-infection while no effect was observed in HeLa wild type cells (**Figure 7B**). This indicates that NF-κB signaling, which is upregulated at a very early time of infection in ZC3-KO cells, cannot be efficiently blocked in the absence of the ZC3H11A protein. This would create conditions where ZC3H11A would have a proviral effect on adenovirus growth.

## Discussion

ZC3H11A is a stress-induced protein that plays an important role in regulating the nuclear mRNA export under situations of stress, such as heat-shock and viral infections (37). Not much is known about its function under immunological stress and role in regulation of immune responses. In this study we have shown that the loss of ZC3H11A protein expression resulted in upregulation of several innate and inflammatory genes (**Graphical Abstract** and **Figures 1**-**3**) associated with activation of the NF-κB transcription factor (**Figure 2A**) due to flawed IκBα mRNA export, suggesting ZC3H11A’s negative regulatory effect on the NF-κB signaling pathway. This findings accentuate a previously unknown mechanism of regulation of NF-κB activity by ZC3H11A.

Mechanistically the enhancement of the NF-κB pathway in ZC3H11A deficient cells appears to be due to a failure to produce sufficient amounts of the IκBα inhibitory protein to sustain the negative feedback loop blocking the NF-κB transcription factor. Transcription of the IκBα mRNA, which is under the control of the NF-κB transcription factor, will lead to the synthesis of the IκBα protein that will translocate to the nucleus where it binds to NF-κB, leading to its dissociation from DNA and export to the cytoplasm, thereby returning it to its inactive form. In cells devoid of ZC3H11A we observed a dramatic reduction in IκBα protein accumulation (**Figures 5C, E**), and an increased accumulation of the NF-κB p65 subunit in the nucleus (**Figure 5A**), suggesting that the active NF-κB transcription factor was retained in the nucleus of the ZC3-KO cells. Interestingly, the failure to produce adequate amounts of the IκBα inhibitory protein in ZC3-KO cells appears to correlate with increased retention of the IκBα mRNA in the cell nucleus (**Figure 6C**). This result is in line with our previous demonstration that ZC3H11A is required for efficient nuclear export of some viral mRNAs, during an adenovirus infection (37). Since ZC3H11A is part of the TREX complex, these results are consistent with the hypothesis that IκBα protein expression is reduced in ZC3H11A depleted cells because of a primary defect in IκBα mRNA export.

In contrast to IL-6 and TNF-α, IL-8 was not upregulated in unstimulated ZC3-KO cells (**Figure 2E**). However, following IL-1ß stimulation the IL-8 mRNA expression was enhanced in ZC3-KO cells compared to wild type HeLa cells. The difference between the response of IL-6 and IL-8 to the loss of ZC3H11A function could be explained by a different signaling requirement of IL-8, which may not rely solely on NF-κB in HeLa cells. IL-8 has been shown to require MAPK signaling in different cell types, including HeLa cells (57).

The increase in IL-6 mRNA expression in ZC3-KO cells is likely, in part, due to increased NF-κB transcription factor binding to the IL-6 promoter (**Figure 4A**). IL-6 expression can also be regulated by other transcription factors, such as NF-IL-6 and AP1 (58). However, our data clearly demonstrate that protein binding to the NF-IL-6 and AP1 binding sites is not enhanced in ZC3H11A-deficient cells. Accumulation of the NF-κB p65 subunit in the ZC3-KO cell nucleus (**Figure 5A**) further support formation of a transcriptionally active NF-κB factor in ZC3H11A-deficient cells. Taken together, our results suggest that the upregulation of the NF-κB pathway in ZC3-KO cells results, at least in part, from an increase in IL-6 promoter activity.

ZC3H11A has been shown to bind to the so-called SOX-resistant element (SRE) in the 3’ UTR of the IL-6 mRNA (54). During a lytic Kaposi’s sarcoma-associated herpesvirus (KSHV) infection, the viral nuclease SOX degrades most cytoplasmic mRNAs. However, the IL-6 mRNA is resistant to SOX cleavage because more than 20 cellular proteins bind to the SRE, some of which have a protective effect against SOX cleavage. It was not tested in this study what effect ZC3H11A had on SOX cleavage. Indeed, our results show that ZC3H11A has a destabilizing effect on the IL-6 mRNA. Thus, the lack of ZC3H11A protein expression resulted in a substantial increase in IL-6 mRNA stability (**Figures 4D, C**). The IL-6 3’ UTR is a crucial element for this destabilizing effect on the IL-6 mRNA, since the GFP reporter gene fused to the IL-6 3’ UTR gave higher reporter protein accumulation in ZC3H11A deficient cells compared to control cells (**Figure 4E**). We further showed that loss of ZC3H11A function induces primary human monocyte differentiation into imDCs (**Figure 3B**). This induction is similar to what has been reported earlier for mice deficient for IκBα that shows an increase of granulocyte/erythroid/monocyte/macrophage colony-forming units (CFU-GEMM), suggesting that the classical NF-κB pathway contributes to the differentiation of these lineages (59–61). In another study, NF-κB was shown to play a major role in the maturation of human dendritic cells induced by nickel sulfate NiSO_4_ (62). Thus, blockage of NF-κB signaling suppressed the NiSO_4_ -induced expression of both CD40 and HLA-DR expression and cytokine production. However, the blockage of NF-κB only partially inhibited NiSO_4_ -induced CD86 and CD83 expression (62). Our results further confirmed that the inhibitory effect of ZC3H11A on IL-6 mRNA accumulation in HeLa cells were also reproduced in the imDCs after ZC3H11A knockdown (**Figure 3A**). Knocking down ZC3 alone was insufficient to enhance basal IL6 mRNA expression in imDCs but only observed after TNF-α and IL-1ß stimulation, in contrast to what we experienced in Hela cells (**Figure 2G**). That could be explained by the different tuning of NF-κB signaling in cancer cells compared to primary cells (63).

It is not clear whether ZC3H11A has an intrinsic ribonuclease activity, like the ZC3H12A protein that also regulates IL-6 mRNA stability (23, 64), or whether ZC3H11A binds to the IL-6 3’ UTR and recruits cellular protein(s) controlling IL-6 mRNA stability. For example, tritestraprolin (TTP, also known as ZFP36), which like ZC3H11A, is a CCCH-type ZnF protein that binds to IL-6 3’ UTR and recruits proteins involved in mRNA decapping and deadenylation, thereby sending the IL-6 mRNA for destruction (23, 65, 66). It is possible that ZC3H11A interactions with these and/or other cellular proteins control the stability of the IL-6 mRNA and potentially other inflammation-related mRNAs.

In our previous work, we identified ZC3H11A as a proviral protein that supported replication of nuclear replicating viruses, like adenovirus, HIV, HSV-1, and influenza virus (37). Interestingly, the safeguard function of ZC3H11A on NF-κB activation and on immune stress and interferon-mediated anti-viral responses might be what nuclear-replicating viruses take advantage of to replicate more efficiently in infected cells. Thus, the retention of the IκBα mRNA in the nucleus results in hyperactivation of NF-κB signaling, something that has severe negative effects on multiple virus infections (66). Adenovirus has a complex relation to NF-κB. The viral E3 transcription unit has two NF-κB binding motifs, required for high E3 promoter activity in lymphoid cells (67, 68). E3 in turn encodes for many of the viral proteins implicated in immune evasion by modulating intracellular signaling events, function of cell surface receptors and secretion of NF-κB-dependent pro-inflammatory cytokines (69, 70). For example, the E3-RIDα protein suppresses NF-κB activation during an acute infection. Thus, E3-RIDα expression blocks the EGF receptor-induced NFκB signaling axis in TNF-α stress-activated cells (69, 70). Because of the importance of the NF-κB signaling pathway in an adenovirus infection it is conceivable that the reason that the steady-state amount of ZC3H11A increases three-fold during an adenovirus infection (37) could be a way for the virus to further suppress NF-κB signaling, to aid virus replication.

Collectively, our findings show a previously unidentified mechanism of ZC3H11A in negative modulation of NF-κB activity and have further progressed the understanding of the regulation of the NF-κB signaling. While in this study we have elucidated one mechanism, other immuno-regulatory mechanisms are still unknown and requires further exploration. ZC3H11A can be harnessed as a therapeutic tool in homeostatic control of aberrant inflammation. It also have implications to be an antiviral target since several viruses exploit ZC3H11A mediated suppression of NF-κB activity and mRNA export for their effective growth.

## Data availability statement

The data presented in the study are deposited in the ncbi repository, accession number PRJNA892750 (https://www.ncbi.nlm.nih.gov/sra/?term=PRJNA892750).

## Ethics statement

Ethical review and approval was not required for the study on human participants in accordance with the local legislation and institutional requirements. Written informed consent for participation was not required for this study in accordance with the national legislation and the institutional requirements.

## Author contributions

MD designed experiments, conducted experiments, analyzed the data and wrote the manuscript. SY conducted the RNA-seq experiments, performed data analysis and helped editing the manuscript along with figures. ZH conducted experiments. CJ and AA designed and conducted experiments. SG reviewed and suggested experiments and edited the manuscript. TP designed plasmid cloning and helped editing the manuscript. SG, ME, and LA participated in constructive discussions and helped editing the manuscript. GA designed experiments, analyzed the data and wrote the manuscript. All authors contributed to the article and approved the submitted version.

## Acknowledgments

We are grateful to Jianxiang Wang, and Dr Wael Kamel who participated at the early stages of this project. We also thank Anette Carlsson for technical assistance. This work was supported by generous grants from the Knut and Alice Wallenberg Foundation (2017-0071); the Swedish Cancer Foundation (180599); and the Swedish Research Council (2017-01592).

## Conflict of interest

The authors declare that the research was conducted in the absence of any commercial or financial relationships that could be construed as a potential conflict of interest.

## Publisher’s note

All claims expressed in this article are solely those of the authors and do not necessarily represent those of their affiliated organizations, or those of the publisher, the editors and the reviewers. Any product that may be evaluated in this article, or claim that may be made by its manufacturer, is not guaranteed or endorsed by the publisher.
