# Supplementary figures and images for: ZC3H11A loss of function enhances NF-κB signaling through defective IκBα protein expression
Source: Front Immunol. 2022 Nov 9;13:1002823. doi: 10.3389/fimmu.2022.1002823 (PMC9681899; doi:10.3389/fimmu.2022.1002823)

Figure S1

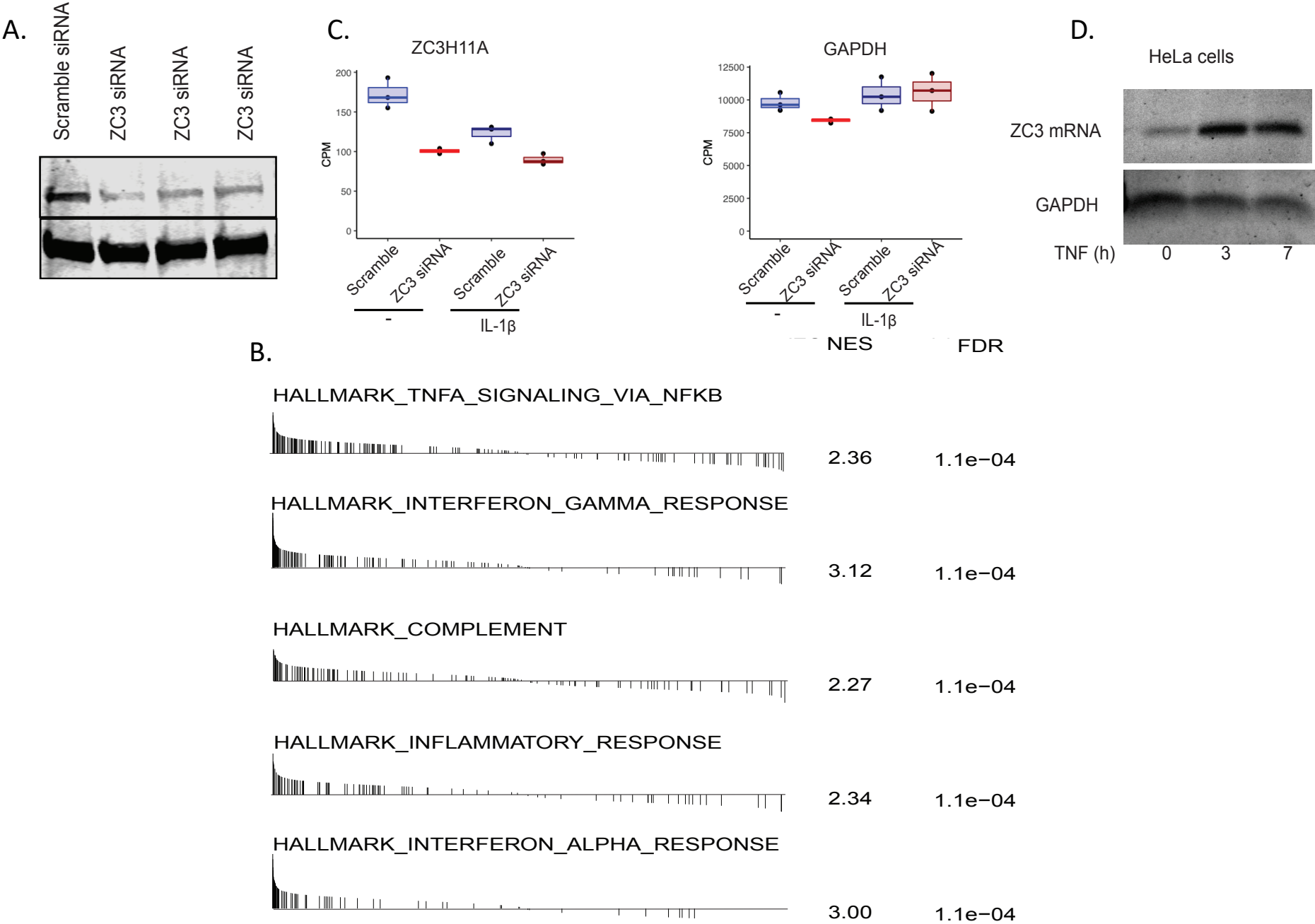

Supplement: Supplementary file 1 [file DataSheet_1.pdf]
