# Supplementary material for: ZC3H11A loss of function enhances NF-κB signaling through defective IκBα protein expression
Source: Front Immunol. 2022 Nov 9;13:1002823. doi: 10.3389/fimmu.2022.1002823 (PMC9681899; doi:10.3389/fimmu.2022.1002823)
Supplement: Supplementary file 2 [file Table_1.docx]

| **Cover LetterPrimer name** | **Sequence** |
| --- | --- |
| Human IL6 (qPCR) | Forward- 5´-CACAGACAGCCACTCACCTC-3`  Reverse- 5´-TTTTCTGCCAGTGCCTCTTT-3` |
| Human IL8 (qPCR) | Forward- 5´-ATGACTTCCAAGCTGGCCGTGGCT-3` Reverse- 5`-TCTCAGCCCTCTTCAAAAACTTCTC-3` |
| Human Ikbα (qPCR) | Forward- 5´-CCTTCCTCAACTTCCAGAACAACC -3` Reverse- 5`-GGCTAAGTGTAGGCAGGTGTGGC-3` |
| Human TNF (qPCR) | Forward- 5´-GCTTGTTCCTCAG CCTCTTCT- 3`  Reverse- 5´-GGTTTGCTACAACATGGGCTA-3` |
| Human IFI27 (qPCR) | Forward- 5´-GTGCCCATGGTGCTCAGT-3`  Reverse-5´- CCAGTGACTGCAGAGTAGCC-3` |
| OAS2 (qPCR) | Forward- 5′-ACCCGAACAGTTCCCCCTGGT-3′  Reverse- 5′-ACAAGGGTACCATCGGAGTTGCC-3′ |
| IFIT1 (qPCR) | Forward- 5′-TCAGGTCAAGGATAGTCTGGAG-3′  Reverse-5´-AGGTTGTGTATTCCCACACTGTA-3′ |
| GAPDH (qPCR) | Forward- 5′-AGAAGGCTGGGGCTCATTTG-3′  Reverse-5´- AGGGGCCATCCACAGTCTTC-3′ |
| ZC3H11A (qPCR) | Forward- 5′-AAGGAAGGACTTACCCATTTTGATATT-3′  Reverse-5´- TGGGTCAGATTTCCCTATGAGAA-3′ |
| IFIT2 (qPCR) | Forward- 5′-GGAGGGAGAAAACTCCTTGGA-3′  Reverse-5´-GGCCAGTAGGTTGCACATTGT-3′ |
| IKBα FISH probe | 5′GCCTTCAGGATGGAGTGGAGGTGCGGGGTGGTGCAGGACTGAGTCAGGACTCCCACGCTGGCCAGGCAGCCCTGC-3′ |
| IL6 FISH probe | 5′CTCCCTGGGCCACACACCCCTCCCTCACACAGGGCTCGACCGCCGCTGGCAGTTCCAGGG-3′ |
| IKBα Northern probe | 5′GGCCAGGTCTCCCTTCACCTGGCGGATCACTTCCATGGTCAGTGCCTTTTCTTCATGGATGATGGCCAAGTGCAGGAACGA-3′ |

**Supplementary Table S1: Sequence of oligonucleotides**
