# Supplementary material for: ZC3H11A loss of function enhances NF-κB signaling through defective IκBα protein expression
Source: Front Immunol. 2022 Nov 9;13:1002823. doi: 10.3389/fimmu.2022.1002823 (PMC9681899; doi:10.3389/fimmu.2022.1002823)
Supplement: Supplementary file 3 [file Table_2.docx]

**Supplementary Table S2: Details about antibodies used**

| **Primary antibody** |  |
| --- | --- |
| Anti-phospho-IκBα (Ser32) | (Cell Signaling mAb #2859) |
| Anti-phospho-IκBα (Ser32) | (Cell Signaling #9242) |
| Anti-NF-κB p65 XP® | Cell Signaling )Rabbit mAb #8242 |
| Anti- GFP | Abcam(ab13970) |
| Anti-Lamin B1 | Abcam (ab16048) |
| Anti-HAdV capsid | Abcam (ab6982) |
| Anti-Actin | Proteintech Cat No. 66009-1-Ig |
| Anti-tubulin | Proteintech Cat No. 11224-1-AP |
| CD14 Antibody, anti-human, APC, REAfinity^TM^ | Miltyni Biotic 130-110-520 |
| Anti-CD80 (B7-1) Monoclonal Antibody (16-10A1) | Thermo Fisher Scientific Catalog # 11-0801- 82 |
| Anti-CD86 | Abcam (ab239075) |
| Anti-CD1a [SK9] | Abcam (ab269357) |
